# B Cells Promote Tumor Progression via STAT3 Regulated-Angiogenesis

**DOI:** 10.1371/journal.pone.0064159

**Published:** 2013-05-29

**Authors:** Chunmei Yang, Heehyoung Lee, Sumanta Pal, Veronica Jove, Jiehui Deng, Wang Zhang, Dave S. B. Hoon, Mark Wakabayashi, Stephen Forman, Hua Yu

**Affiliations:** 1 Department of Cancer Immunotherapeutics and Tumor Immunology, Beckman Research Institute, City of Hope Comprehensive Cancer Center, Duarte, California, United States of America; 2 Department of Medical Oncology, Beckman Research Institute, City of Hope Comprehensive Cancer Center, Duarte, California, United States of America; 3 Department of Molecular Oncology, John Wayne Cancer Institute, Santa Monica, California, United States of America; 4 Department of Surgery, Beckman Research Institute, City of Hope Comprehensive Cancer Center, Duarte, California, United States of America; 5 Department of Hematopoietic Cell Transplantation, Beckman Research Institute, City of Hope Comprehensive Cancer Center, Duarte, California, United States of America; 6 Center for Translational Medicine, Shanghai Zhangjiang High-Tech Park, Shanghai, China; National Cancer Institute (INCA), Brazil

## Abstract

The role of B cells in cancer and the underlying mechanisms remain to be further explored. Here, we show that tumor-associated B cells with activated STAT3 contribute to tumor development by promoting tumor angiogenesis. B cells with or without Stat3 have opposite effects on tumor growth and tumor angiogenesis in both B16 melanoma and Lewis Lung Cancer mouse models. *Ex vivo* angiogenesis assays show that B cell-mediated tumor angiogenesis is mainly dependent on the induction of pro-angiogenic gene expression, which requires Stat3 signaling in B cells. Furthermore, B cells with activated STAT3 are mainly found in or near tumor vasculature and correlate significantly with overall STAT3 activity in human tumors. Moreover, the density of B cells in human tumor tissues correlates significantly with expression levels of several STAT3-downstream pro-angiogenic genes, as well as the degree of tumor angiogenesis. Together, these findings define a novel role of B cells in promoting tumor progression through angiogenesis and identify STAT3 in B cells as potential therapeutic target for anti-angiogenesis therapy.

## Introduction

The type and density of immune cells in the tumor tissue have recently been shown to be one of the most reliable parameters for predicting a patient’s clinical outcome in certain types of cancer [1]–[4]. For example, the density of T cells in colorectal tumor tissues represents a better prognostic indicator for patient outcome than current staging systems [2], [3]. Relevant to this, higher infiltration of regulatory T cells or myeloid-derived suppressor cells predicts poor survival in other types of cancer patients, whereas the massive infiltration of CD8^+^ T cells or M1 macrophages in tumor tissue is strongly associated with favorable patient outcome [5]–[9].

B cells are prevalent in tumor tissues of various human cancers, and found in aggregates with other immune cells, mainly at inflammatory sites [10]. Intriguingly, contrary to the common notion that humoral and cellular immune responses work in opposition, the presence of B cells together with CD8^+^ T cells in tumor tissues has been correlated with better patient survival than in tumor tissues with either cell alone [1], [11]. Moreover, B cell-mediated antibody production against tumor antigens is associated with better clinical outcome in human medullary breast carcinoma [12]. While these studies demonstrate the beneficial effect of B cells on anti-cancer immunity, a cancer-promoting role of B cells has also been recognized.

Important studies in mouse skin cancer models have revealed that B cells are required for *de novo* carcinogenesis, in which increased immunoglobulin deposition by B cells in premalignant skin initiates the recruitment of other immune cells [13]. These events in turn induce the activation of FCγ receptors (FCγRs), leading to chronic inflammation and promotion of malignant progression [14], [15]. Importantly, it has also been demonstrated that infiltration of B cells due to androgen ablation induces the production of lymphotoxin to promote castration-resistant prostate cancer [16]. Moreover, in human cancers, B cell-mediated production of immune complexes in the circulation or in the tumor tissue does not always confer protection against tumor antigens but rather correlates with poor clinical outcome in certain cancer patients [4], [17]. Supporting a role of B cells in promoting cancer progression is the observation that adoptive transfer of B cells into B- and T-cell deficient mice restores malignant characteristics in mouse tumors, such as tumor vasculature [13]. Furthermore, the degree of B cell infiltration is a predictor of patient survival and correlates highly with activated STAT3 [18]. However, the underlying molecular mechanisms on B cell-mediated tumor development are unclear.

Angiogenesis is a hallmark of cancer and anti-angiogenesis therapies have shown promise for treating cancer [19]–[22]. Tumor angiogenesis requires the interplay between tumor cells and tumor-infiltrating stromal cells [23]–[26]. Several reports show that signal transducer and activator of transcription 3 (STAT3) is crucial for tumor angiogenesis [27]–[29]. Our recent studies have also demonstrated that STAT3 mediates multidirectional crosstalk among tumor cells, endothelial cells and myeloid cells in promoting tumor angiogenesis [30]. In the current study, we define a crucial role of B cells as well as their STAT3 activity as important contributors for tumor progression and tumor angiogenesis.

## Materials and Methods

### Ethics Statement

The study on human tissue array slides and human prostate tumor tissues was approved by the City of Hope Institutional Review Board (COH IRB 09213). Human melanoma tumor and normal skin tissue sections were provided by John Wayne Cancer Research Institute (JWCI), with approval from JWCI and Western Institutional Review Board (WIRB 1095596). Informed consent was waived by the IRB because the research was performed on de-identified archival tissues.

Mouse care and experimental procedures were carried out under pathogen-free conditions in accordance with established institutional guidance and approved protocols from the Institutional Animal Care and Use Committee of Beckman Research Institute at City of Hope Medical Center.

### Materials

The B16 mouse melanoma cell line and MB49 mouse bladder cancer cell line were obtained from American Type Culture Collection (ATCC). The Lewis lung carcinoma (LLC) cell line was obtained from L. Wu (University of California, Los Angeles). Mouse endothelial cell lines derived from prostate were kindly provided by S. Huang and I. Fidler (M.D. Anderson Cancer Center, Houston, Texas)[31]–[33]. The C4 mouse melanoma cell line was kindly provided by I. Fidler (University of Texas M.D. Anderson Cancer Center). Tumor conditioned medium (TCM) was prepared from C4 cells as described [34]. All cells were maintained in RPMI 1640 or DMEM medium supplemented with 5%–10% FBS.

### Animals

*Stat3* ^flox^ mice were provided by S. Akira (Osaka University, Suita, Osaka, Japan) and K. Takeda (Kyushu University, Fukuoka, Japan). *Rag1^−/−^(*ko)Momj/B6.129S7 mice were purchased from the Jackson Laboratory. *Stat3* ^flox^ and *Mx1*-*Cre* or *CD19-Cre* mice were crossed and treated with polyinosinic-cytidylic acid to obtain *Stat3* conditional knockouts in the hematopoietic system or in B cells. C57BL/6 mice were purchased from the National Cancer Institute (Frederick, MD).

### *In vivo* Tumor Experiments

To obtain tumor-primed B cells, B16, MB49 or LLC tumor cells (1 to 2×10^5^) were first implanted subcutaneously into the flank of C57BL/6 mice with *Stat3* ^+/+^ and *Stat3^−/−^* hematopoietic cells, which is generated by crossing *Stat3* ^flox^ and *Mx1*-*Cre* mice. Spleen, tumor-draining lymph nodes (TDLN) as well as tumor specimens were harvested after 14 days and processed further to isolate B cell populations for RNA and protein extraction. For co-implanting tumor cells with B cells into *Rag1* ^−/−^ mice, B cells isolated from spleen of tumor-bearing mice (1×10^6^) were mixed 10∶1 ratio with either B16 or LLC tumor cells then injected into *Rag1* ^−/−^ mice. Tumor size was measured every other day for the indicated time. Tumors were harvested then pooled to prepare frozen tissue sections for immunofluorescent staining. Tumor-infiltrating B cells were also isolated from pooled tumors to prepare RNA and protein for real-time RT-PCR and western blotting, respectively.

To generate experimental lung metastasis model, B16 tumor cells (5×10^5^) were injected intravenously into C57BL/6 mice with *Stat3^+/+^* or *Stat3^−/−^* B cells, which is generated by crossing *Stat3* ^flox^ and *CD19*-*Cre* mice. After 15 d, lungs were removed and washed in Hank’s buffered salt solution (HBSS). Number of visible metastatic tumor nodules was enumerated by counting individual nodules. B16 tumor nodules were easily identifiable due to their pigmentation.

### B Cell Preparation

To isolate tumor-infiltrating B cells, tumors were gently minced and incubated (30 min, 37°C) with collagenase D and DNAse solution (Roche, 400 U/ml). Cells were resuspended by repeated pipetting and filtered through a mesh filter. Mononuclear cells were separated by gradient centrifugation using Histopaque (Sigma, 1.083 g/ml) and kept as tumor-infiltrating immune cells. Then tumor B cells were isolated from immune cell mixtures using the Mouse CD19 Positive Selection Kit (EasySep, StemCell Technologies) or MACS Cell Separation System Positive Selection Kit (Miltenyi Biotec). B cells from spleens and lymph nodes were prepared in the same manner.

### Immunofluorescence and Immunohistochemistry (IHC) Staining

For immunofluorescent staining, the flash-frozen tumor specimens or frozen Matrigel plugs were fixed in formaldehyde and permeabilized with methanol before antibody staining. After blocking, sections were stained with primary antibody overnight followed by incubation with a secondary antibody, mounted in Vectashield mounting medium containing 4′6′-diamidino-2-phenylindole (DAPI) (Vector Laboratories). In some cases, sections were stained with Hoechst 33342 (1∶200) to visualize nuclei then mounted in Mowiol coverslip mounting solution. Images were taken by confocal microscopy using CLSM510Meta confocal microscope (Zeiss). Cells expressing either CD19 B cell markers or p-STAT3 were enumerated from ten microscopic fields with at least 1,000 cells by Image Pro 6.3 software.

For IHC, paraffin tissue slides were deparaffinized, rehydrated through an alcohol series and autoclaved in Antigen Unmasking Solution (Vector Laboratories). After wash, tissue sections were treated with 1% H_2_O_2_ in methanol for 10 min at room temperature, then incubated with the primary antibody for overnight at 4°C and subjected to ABC/DAB detection method (Vector Laboratories). The expression level of primary antibody in tumor tissues was visualized by a Nikon ECLIPSE TE2000-U microscope and imaged using SPOT software.

The primary antibodies used are anti-pY705-STAT3 (Santa Cruz Biotechnology Inc. or Cell Signaling), anti-CD19, a marker for human B cells (AbD Serotec), anti-B220, mouse B cell marker (eBioscience), anti-MMP9 (Cell Signaling) and anti-CD31 for human and mouse blood vessels (Santa Cruz Biotechnology Inc. and BD Pharmingen, respectively).

### Tube Formation Assay

Endothelial cells (ECs) and mouse B cells with or without *Stat3* were co-cultured on neutralized collagen at 1∶1 ratio in 1% FBS-RPMI 1640 medium (1.2 mg/ml; BD Biosciences) for 16 h. The cells were fixed in 4% paraformaldehyde for 10 min, washed, and analyzed under an inverted light microscope (Nikon). Closed networks of vessel-like tubes were counted from each well. For antibody neutralization studies, B cells were co-incubated with ECs in the presence of either anti-IgG or anti-Vegf antibodies (5 µg/ml; R&D Systems).

### *In vivo* Matrigel Angiogenesis Assay

B cells from C57BL/6 mice with *Stat3^+/+^* and *Stat3^−/−^* hematopoietic cells (*Stat3* ^flox/flox^ and *Stat3* ^flox/flox^-*Mx1*-*Cre* mice) were mixed with tumor cells in growth factor-reduced Matrigel (BD Biosciences) at 10∶1 ratio then implanted subcutaneously into the flank of *Rag1^−/−^* mice. After 6 days, Matrigel plugs were photo-imaged with Cannon SX200IS digital camera then dissected to analyze hemoglobin content using Drabkin reagent (Sigma-Aldrich).

### Transwell Migration Assay and B Cell Proliferation Assay

For EC migration, collagen-coated inserts with 8 µm pore size (Corning-Costar, Cat. 3422) were used. Cells (1.5×10^6^) were placed in the top chamber of the insert, and the bottom well was filled with or without 10% tumor conditioned medium (TCM) or B cells with *Stat3* ^+/+^ and *Stat3^−/−^*. After 6 h, the inserts were removed, and the inner side was whipped with cotton swaps and stained with Harris hematoxylin solution (Sigma-Aldrich). After washing, filters were cut out, mounted on microscope slides. Four images covering the majority of the sample were collected from each filter, then cells were counted using ImageJ software. Migrated B cells were counted by flow cytometry.

For CFDA-SE labeling (Invitrogen), 5×10^5^ B16 tumor-primed B cells were stained with CFSE (0.5 µM, final concentration) for 15 min at 37°C and plated in 2% FBS-RPMI 1640 with or without additional 10% TCM in the transwell chamber. After 24 hrs, B cell migration and proliferation were determined by flow cytometry.

### RNA Isolation and Quantitative Real-time PCR

Total RNA was extracted using the RNeasy kit (Qiagen) or RNA queous-Micro Scale RNA Isolation kit (Ambion) according to the manufacturer’s instruction. RNA (0.5 to 1 µg) was reverse-transcribed to cDNA using iScript cDNA Synthesis Kit (Bio-Rad), and real-time PCR reactions were performed using iQ SYBR Green supermix (Bio-Rad) on a DNA Engine thermal cycler equipped with Chromo4 detector (Bio-Rad). Gene specific primer sets were purchased from SA Bioscience. The 18S rRNA housekeeping gene was used as an internal control to normalize mRNA expression.

### Protein Preparation and Western Blot Analysis

Cells or tissues were lysed in a modified RIPA buffer containing 50 mM Tris, pH 7.4, 1% NP-40, 150 mM NaCl, 1 mM EDTA, 1 mM Na_3_VO_4_ and protease inhibitor cocktail (Roche). Tissue lysates were prepared by FastPrep homogenizor (MP Biomedicals). The lysates were clarified by centrifugation, and protein concentrations were determined by Bio-Rad protein assay. Equivalent amounts of total cellular proteins were separated by SDS plus 8–15% PAGE according to protein molecular weight, transferred onto nitrocellulose membranes, probed with the respective antibodies, and detected for signals using horseradish peroxidase-conjugated secondary antibodies by enhanced chemiluminescence (Thermo Scientific). Antibodies recognizing p-STAT3 (Y705), STAT3, S1PR1 (clones H-60 and A-6), VEGF (A-20) were purchased from Santa Cruz Biotechnology Inc.; FGF2 was from BD Transduction Lab; others were p-STAT3 (Y705) (Cell Signaling), HIF-1α (Novus Biologicals), MMP9 (Cell Signaling) and β-actin (Sigma).

### Statistical Analysis

For the study of in vivo mouse tumor growth, two-way ANOVA and Bonferroni post-test were used to calculate differences. One-way ANOVA or unpaired t-test was used to calculate P values in all other cases. P values are shown in figures and legends. Data were analyzed using Prism software (GraphPad Software, Inc.). Data were shown as means ± SEM, unless indicated otherwise.

## Results

### B Cells with Activated Stat3 Increase Tumor Growth *in vivo* by Enhancing Tumor Angiogenesis

**Figure 1:**
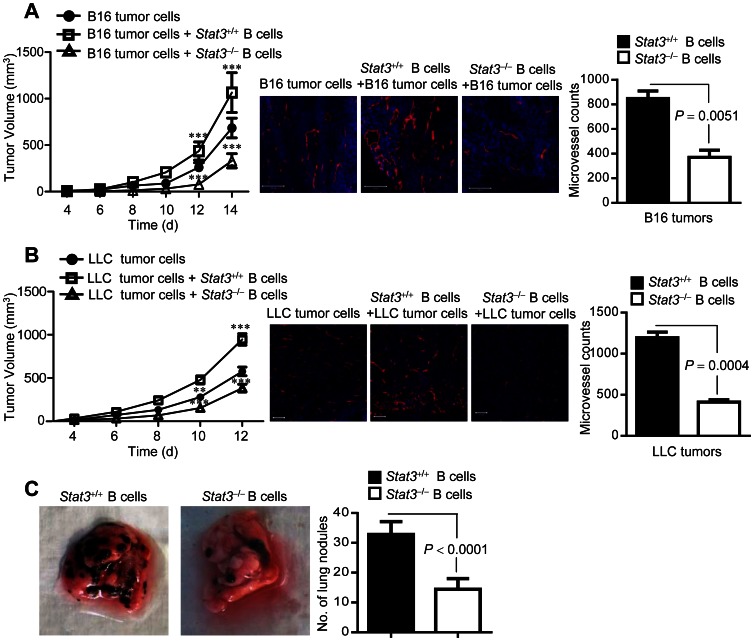
B cells with activated Stat3 accelerate tumor progression and increase blood vessel formation in tumors. (**A and B**) Left, Growth curve of B16 (**A**) or LLC (**B**) tumors in *Rag1^−/−^* mice, without or with *Stat3^+/+^*or *Stat3* ^−/−^ B cells. B cells were enriched from splenocytes of B16 or LLC tumor-bearing mice with or without *Stat3* ablated in hematopoietic cells; *n* = 8; ***, *P*<0.001. Middle, Immunofluorescent staining of tumors to detect microvessels (anti-CD31, red) and nuclei (Hoechst, blue); scale bars, 200 µm. Right, Graph showing the number of CD31^+^ vessels in the tumor tissues (3 microscopic fields/section); means ± SD, *n* = 2. (**C**) Representative images (left) and a graph (right) showing the number of spontaneous lung metastatic nodule in mice with *Stat3^+/+^* or *Stat3^−/−^* B cells receiving B16 tumor cells; means ± SEM, *n = *5.

*Stat3* ablation in hematopoietic cells or treatment with CpG-*Stat3* siRNA efficiently abolishes Stat3 activity in myeloid cells and B cells, leading to reduction of tumor burden and/or metastasis in mice [35], [36]. While myeloid cells and their intrinsic Stat3 signaling have been demonstrated to be important for tumor progression via multiple mechanisms, including angiogenesis [30], [35]–[37], the counterpart effects of *Stat3* ablation in B cells on tumor have not been assessed. In growing tumors, Stat3 is persistently activated in tumor-infiltrating B cells (Figure S1). To further determine whether tumor-associated B cells and their intrinsic Stat3 activity directly contribute to tumor growth *in vivo*, we implanted B16 mouse melanoma cells or LLC mouse lung tumor cells in the presence of either *Stat3^+/+^* or *Stat3^−/−^* B cells into *Rag1^−/−^* mice, which lack mature B or T cells. Results from these experiments showed that addition of *Stat3*-expressing B cells in the tumor microenvironment accelerated tumor growth in both B16 melanoma and LLC mouse lung tumor models (Fig. 1A and 1B, left panels). In contrast, adding *Stat3^−/−^* B cells to the tumor environment reduced tumor growth. Furthermore, the differences in tumor growth caused by Stat3 activity in B cells were accompanied by differential intensities of tumor angiogenesis (Fig. 1A and 1B, middle and right panels). Not only important for promoting tumor growth, *Stat3^+/+^* B cells also accelerate tumor progression through upregulating metastatic potential of B16 tumor cells *in vivo* (Fig. 1C).

### B Cells Induce Endothelial Cell Tube Formation via Stat3

**Figure 2:**
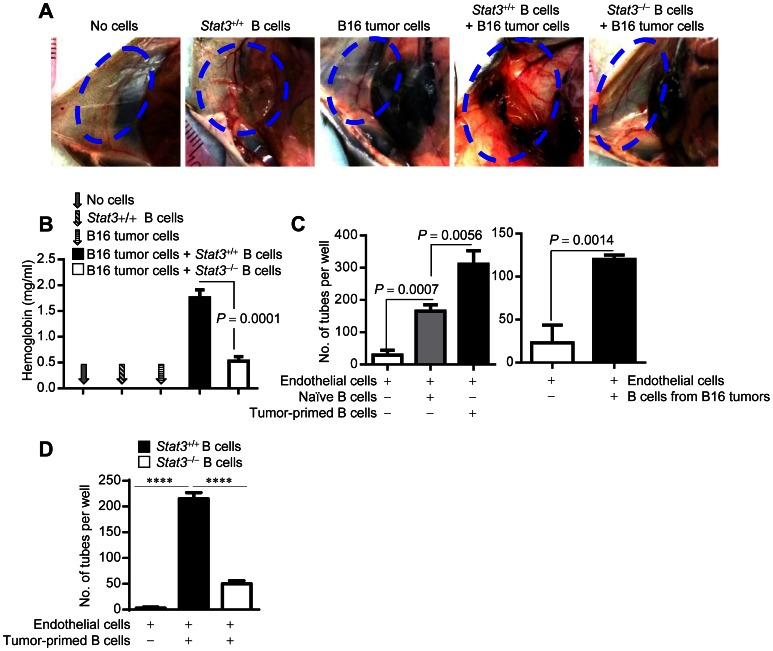
B cells promote tumor angiogenesis by enhancing endothelial cell function in a Stat3-dependent manner. (**A**) Representative images of vessel formation in Matrigel plugs implanted in *Rag1^−/−^* mice. The Matrigel plugs contain either B16 tumor cell alone, *Stat3^+/+^* B cell alone or B16 tumor cells plus *Stat3^+/+^* or *Stat3* ^−/−^ B cells, which were isolated from splenocytes of tumor-bearing mice; *n* = 5; area indicated by blue dot showing level of blood vessel formation. (**B**) Hemoglobin contents in the pooled Matrigel plugs determined by colorimetric assay; means ± SEM, *n* = 5. (**C**) *In vitro* collagen tube formation assay showing the number of tubes formed by ECs with or without the indicated B cells. *Stat3^+/+^* B cells were enriched from splenocytes of B16 tumor-bearing mice (left) or B16 tumors (right); means ± SEM, *n* = 3. (**D**) B cell-mediated endothelial cell tube formation requires Stat3 signaling in B cells. EC tube formation by co-culturing ECs with tumor-primed *Stat3^+/+^* or *Stat3^−/−^* splenic B cells. Tumor-primed B cells were enriched from splenocytes of MB49 tumor-bearing mice with *Stat3^+/+^* or *Stat3^−/−^* hematopoietic cells; means ± SEM, *n* = 3; ****, *P*<0.0001.

To further substantiate the importance of B cells with activated Stat3 in stimulating tumor angiogenesis, we performed *in vivo* Matrigel assays using B cells with or without intact Stat3 signaling. Matrigel plugs containing both tumor cells and *Stat3^+/+^* B cells exhibited markedly increased tumor vascularization *in vivo*, compared to those with only B16 tumor cells or B cells (Fig. 2A and 2B). Although addition of *Stat3^−/−^* B cells to B16 tumor cells increased blood vessel formation somewhat, it was highly significantly less compared to that by adding *Stat3^+/+^* B cells to B16 tumor cells (Fig. 2A, 2B and Figure S2A). Immunofluorescent staining of sections prepared from Matrigel plugs also showed the promoting effect of *Stat3^+/+^* B cells on tumor angiogenesis (Figure S2B). Next, we assessed whether B cells and their intrinsic Stat3 signaling would affect endothelial cells’ ability in forming blood vessels. Co-culturing endothelial cells with naïve splenic B cells significantly enhanced endothelial cell tube formation, indicating that B cells can upregulate the angiogenic potential of endothelial cells (Fig. 2C). Moreover, endothelial cell tube formation was further increased by tumor-primed splenic B cells prepared from B16 tumor-bearing mice, as well as by B16 tumor-infiltrating B cells (Fig. 2C). We also determined whether the tumor milieu-induced, B cell-mediated tube formation was at least in part due to elevated Stat3 activation in B cells. Results from this set of experiments showed that tumor-primed B cells lacking a functional *Stat3* did not effectively support endothelial cells to form tube-like structures (Fig. 2D). In addition to promoting endothelial cell migration (Figure S3A), Stat3 activation intrinsic to B cells was critical for B cells’ own migratory ability to tumor-secreted chemoattractants (Figure S3B, left). However, B cell Stat3 was not essential for the proliferative potential of B cells in the tumor milieu (Figure S3B, right).

### Stat3 Signaling Intrinsic to B Cells is Crucial for B Cell Expression of Pro-angiogenic Genes

**Figure 3:**
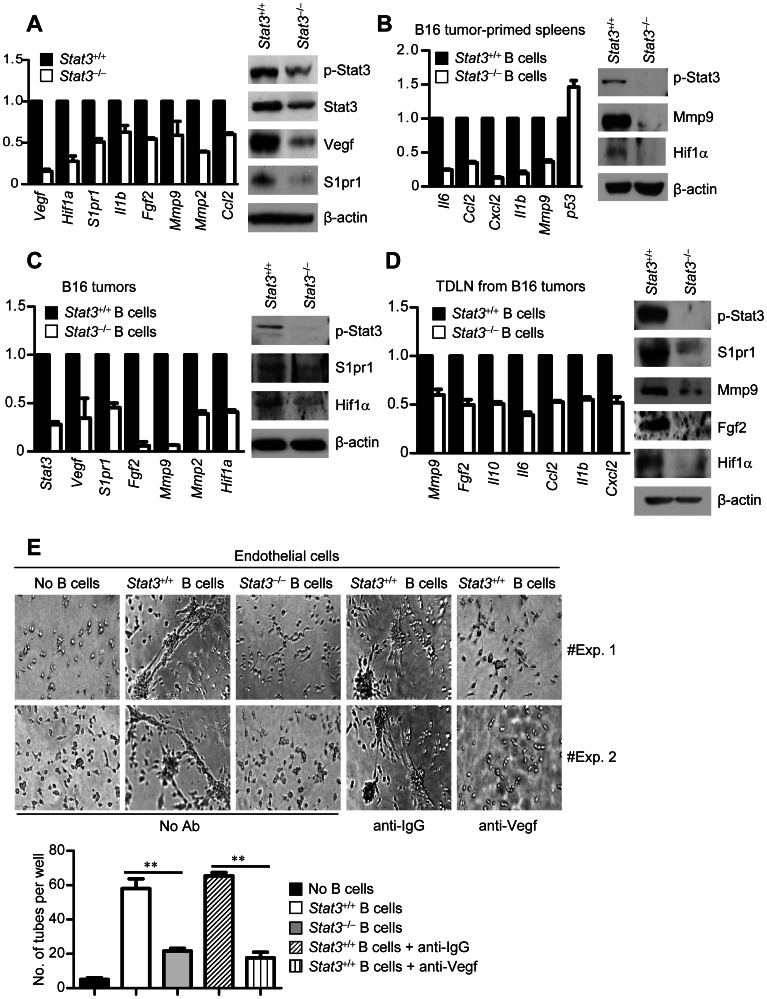
*Stat3* intrinsic to B cells is crucial for expression of pro-angiogenic genes and endothelial cell tube formation. (**A**) Expression levels of mRNA (left) or protein (right) of the indicated genes in B16 tumors. The tumors were formed by implanting B16 tumor cells with the indicated tumor-primed B cells in *Rag1^−/−^* mice. (**B**) mRNA (left) or protein (right) expression levels of the indicated genes in tumor-primed B cells from splenocytes of B16-tumor bearing C57BL/6 mice with *Stat3^+/+^* or *Stat3^−/−^* hematopoietic cells. (**C** and **D**) mRNA and protein levels of the indicated pro-angiogenic genes in *Stat3^+/+^* or *Stat3^−/−^* B cells isolated from either B16 tumors (**C**) or B16 tumor-draining lymph nodes (TDLN) (**D**). In all cases, mRNA is determined by real-time RT-PCR. The relative amount of mRNA is compared to that of *Stat3^+/+^* B cells, which is designated as 1 (means ± SEM, *n* = 3). Western blotting data represent two independent experiments. (**E**) Representative images of EC tube formation by co-culturing ECs with splenic *Stat3^+/+^* B cells from B16 tumor-bearing mice in the presence of VEGF neutralizing antibodies. IgG antibody at the same concentration is used as control; *n* = 3 (top). Quantitative graph showing the average number of vessel-like structures formed by ECs upon addition of the indicated B cells; means ± SEM, *n* = 3; **, *P*<0.001 (bottom).

To identify the molecular events underlying *Stat3^+/+^* B cell-driven tumor angiogeneisis, we assessed whether tumor-associated B cells themselves expressed pro-angiogenic factors in a Stat3-dependent manner. When *Stat3* was functionally ablated in B cells in the tumors, the overall Stat3 activity and expression levels of several angiogenic genes in the whole tumor were decreased (Fig. 3A). Many of the genes involved in angiogenesis shown in Fig. 3A are known to be modulated by Stat3 [38].

We also isolated B cells from tumor-primed splenocytes and assessed expression levels of several angiogenic genes. The results indicated that the expression levels of multiple pro-angiogenic factors in B cells were reduced by functionally ablating *Stat3* (Fig. 3B). Moreover, real-time RT-PCR showed that Stat3 activity promoted expression of these pro-angiogenic genes in B cells isolated from tumor tissues and the tumor draining lymph nodes (Fig. 3C and 3D). Western blot analysis of tumor-associated B cells and B cells from the tumor-draining lymph nodes also confirmed an important role of Stat3 intrinsic to B cells in promoting expression of angiogenic factors in the tumor microenvironment (Fig. 3C and 3D). Furthermore, neutralizing VEGF, a potent angiogenic factor downstream of STAT3, with antibodies efficiently blocks B cell-mediated formation of tube-like structures by endothelial cells (Fig. 3E). Taken together, our studies show that B cells are an important source of angiogenic factors and B cell intrinsic Stat3 activity is crucial for B cell production of pro-angiogenic factors in the tumor milieu.

### B Cells with Activated STAT3 Accumulate in Human Tumors

**Figure 4:**
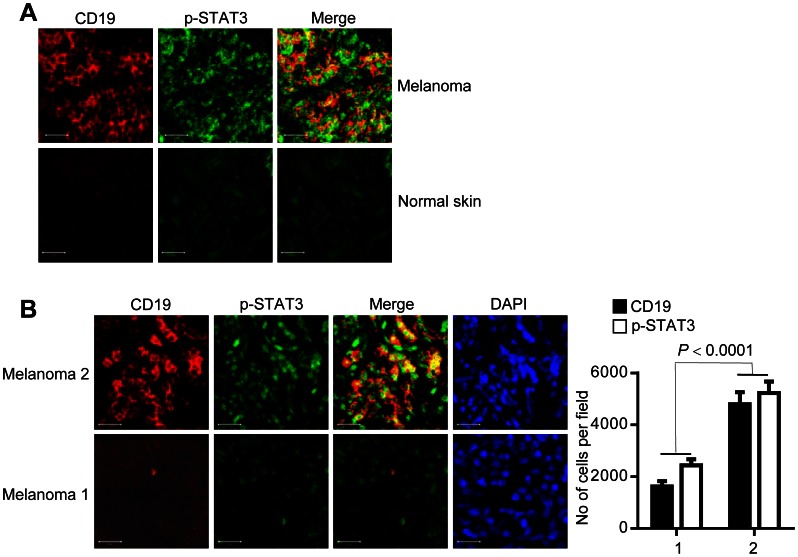
B cells with activated STAT3 accumulate in human tumors. (**A**) Immunofluorescent staining of human melanoma and normal human skin tissue sections; anti-CD19 (red; B cell marker) and anti-p-STAT3 (green). Scale bars, 20 µm. (**B**) B cells in primary tumor sites impact overall tumor STAT3 activity. Confocal microscopic images showing primary melanoma tumor tissue staining of B cells and p-STAT3 (left), with quantification of CD19 and p-STAT3 positive cells (right). Scale bars, 20 µm. Total ten microscopic fields (10 X) were examined for each tumor section; *n* = 2.

We next evaluated in human tumor tissues whether our findings in mouse tumor models that B cells with elevated Stat3 activity are crucial for tumor progression in part via promoting tumor angiogenesis are clinically relevant. We first compared the relative presence of B cells in tissue sections of human melanoma *vs*. human normal skin. Results from these analyses showed that the number of B cells in human melanoma tissues was high relative to normal skin tissues (Fig. 4A). Immunofluorescent staining further revealed the presence of persistently activated STAT3 in B cells infiltrating human melanoma (Fig. 4A). B cell accumulation and STAT3 activity were also highly elevated in all other human tumor types examined, including gastric, lung, liver and prostate cancers (Figure S4A). Moreover, number of cells with activated STAT3 in melanoma tissues was markedly elevated in those tissues with high tumor-associated B cells (Fig. 4B and Figure S4B).

### B Cell Infiltration in Human Tumors is Associated with Tumor Angiogenesis and Upregulation of Pro-angiogenic Genes

**Figure 5:**
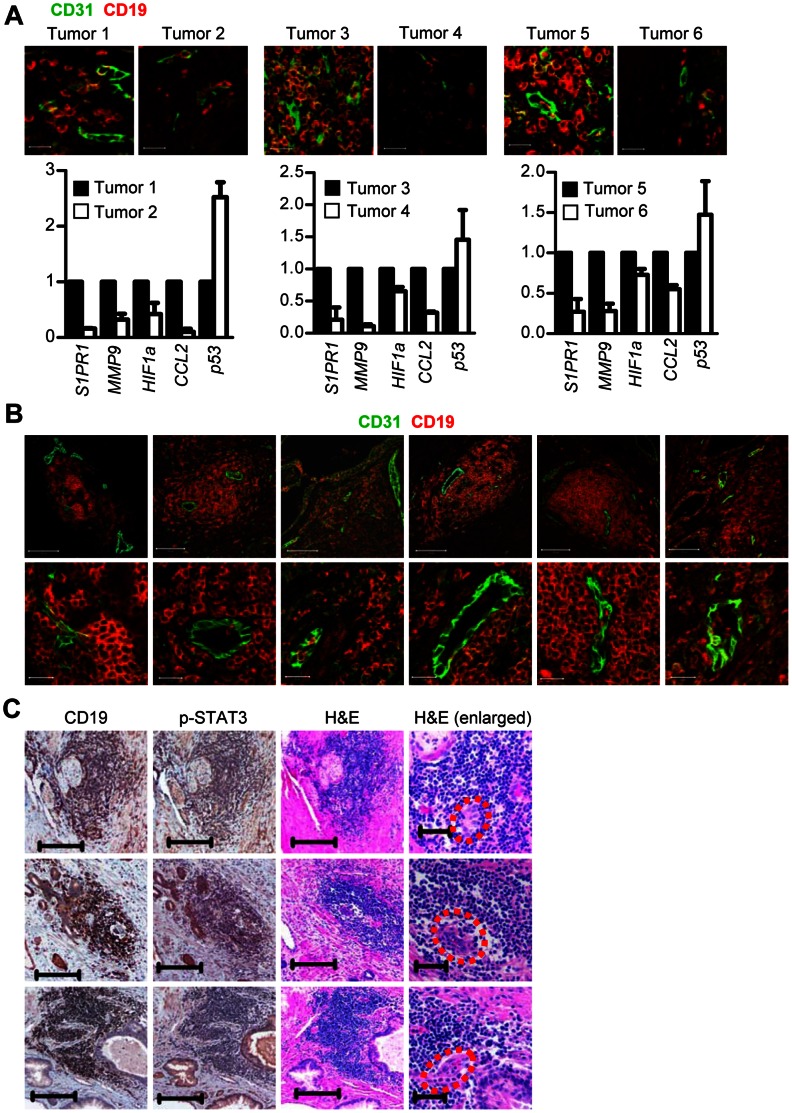
B cells with activated STAT3 express pro-angiogenic genes and accumulate around microvessels in human tumors. (**A**) B cells are important for expression of pro-angiogenic genes within human prostate tumor tissues. The density of B cells around tumor vasculature in prostate tumor tissue was determined by immunofluorescent staining using anti-CD19 and anti-CD31 antibodies (top); scale bars, 20 µm. Real-time RT-PCR measuring RNA expression levels of pro-angiogenic genes in the consecutive human prostate tumor tissue sections (bottom). The relative amount of mRNA is normalized to *18S* and compared to RNA levels in tumor tissues with high p-STAT3, which is designated as 1; means ± SD, *n* = 2. (**B**) Immunofluorescent staining of human prostate tumor tissue sections showing B cells and microvessels with CD31^+^ endothelial cells (green). Scale bars, 100 µm in the original (top) and 20 µm in the enlarged (bottom). (**C**) B cells around the microvessels display persistently activated STAT3. IHC showing B cells and p-STAT3-positive cells in the same area of human prostate tumor tissues; scale bars, 200 µm. H&E staining of the consecutive tissue sections. Microvessel-like structures are marked by red dots; scale bars, 200 µm in the original and 50 µm in the enlarged.

To assess whether B cells could impact on tumor angiogenesis through STAT3-regulated pro-angiogenic genes in human tumors, we first stained human prostate tumor tissues for p-STAT3, CD19 and CD31. We then prepared RNAs from the tumor tissues with differential numbers of p-STAT3-positive B cells. Results from the analysis indicated that expression levels of STAT3-regulated pro-angiogenic genes, such as *S1PR1*, *MMP9* and *HIF1a,* correlated with the density of tumor-infiltrating B cells in human prostate cancers (Fig. 5A). On the other hand, an elevated expression of p53 was associated with lack of B cells in the tumors (Fig. 5A). The important role of p53 in inhibiting tumor angiogenesis and the inhibitory effect of STAT3 on p53 expression has been documented [39]–[41]. By co-staining tumor tissues with antibodies recognizing B cells and blood vessels, we observed that CD19*^+^* B cells had a tendency to accumulate around microvessels rather than distribute evenly throughout human tumor tissues (Fig. 5B and Figure S5). Moreover, B cells around tumor vasculature exhibited persistently activated STAT3 (Fig. 5C).

## Discussion

A crucial role for tumor STAT3 in upregulating proliferation/survival of tumor cells as well as dampening proper function of immune cells such as myeloid cells and T cells has been well characterized [37], [38], [42]. Our study further reveals a previously unrecognized role of B cell STAT3 in accelerating tumor progression through increasing angiogenesis. Since B cells are commonly present as aggregates with other immune cells [10], B cells may contribute to a network with other cells to promote tumor angiogenesis in a STAT3-dependent manner. Supporting this, STAT3 is important for regulating multi-directional feed-forward loop between tumor cells, tumor-associated myeloid cells and endothelial cells for tumor angiogenesis [30]. STAT3 also contributes to T cell-mediated tumor angiogenesis, since inhibiting STAT3 in T cells halts tumor growth in part by inducing collapse of blood vessels [43]. Whether STAT3 in B cells synergistically work with other immune cells including myeloid cells and T cells for tumor angiogeniesis warrants further investigation.

While myeloid cells and activated T cells release pro-angiogenic factors such as VEGF [30], [44], results from our study clearly show that B cells are an important producer of STAT3-donwstream pro-angiogenic factor in the tumor microenvironment. Furthermore, in human tumor tissues as well as in mouse tumors, many of the angiogenic factors secreted by B cells are canonical STAT3 activators, implying a positive feedback loop in tumors. This could partially explain why the density of tumor-infiltrating B cells reflects the overall STAT3 activity in human tumor tissues in our study. Although our study shows that STAT3 is persistently activated in some, but not all of B cells in human cancers, the subset of B cells with activated STAT3 might be sufficient to potentiate and maintain persistent STAT3 activation in tumors. While some report suggest the oncogenic role of B1 regulatory cells in mouse tumor models [45], [46], further studies are required to define the subset of B cells with persistently activated STAT3 in B cell-mediated tumor angiogenesis. Nonetheless, we show that tumor-infiltrating B cells are critical for STAT3 activation and for angiogenic processes in the tumor microenvironment.

STAT3 activation has been linked to several autoimmune diseases, including systemic lupus erythematosus, a condition arising from uncontrolled humoral immune responses [47]–[49]. Conversely, STAT3 activation is absent in diseases characterized by poor humoral immune responses such as hyper IgE syndrome [50]. Furthermore, B cell *Stat3*-deficient mice fail to mount antigen-specific T cell-dependent IgG responses [51], suggesting a complex regulation between B cell-mediated humoral immunity and STAT3. B cell-mediated tumorigenesis in mouse skin tumor models requires activation of FcγR but not complement factors [14]. STAT3 has been implicated in the regulatory circuitry of complement regulatory proteins [51]. Whether humoral components are involved in persistent B cell STAT3 activity in tumors awaits to be determined.

Our findings argue for B-cell direct-targeting approaches to complement current anti-angiogenesis strategies. As one example, we have developed a CpG-conjugated siRNA *in vivo* delivery platform that targets mainly B cells and myeloid cells [35]. Other B cell-directed targeting includes antibody-based approaches [52], [53]. Taken together, we have demonstrated the importance of B cells in promoting tumor progression, and B cells and/or their intrinsic STAT3 activity as targets for anti-angiogenic therapies.

## Supporting Information

Figure S1 **Stat3 is persistently activated in tumor-infiltrating B cells.** Immunofluorescent staining of LLC tumors showing p-Stat3-positive cells (green) and B cells (red). LLC tumors were grown in C57BL/6 mice. Tumor-infiltrating B cells were detected with anti-B220 antibodies.
(TIF)

Figure S2 **B cells promote tumor angiogeneis in a Stat3-dependent manner.** (**A**) Images of vessel formation in Matrigel pugs containing B16 tumor cells and *Stat3^+/+^* or *Stat3^−/−^* B cells; *n*  =  7 (left). Hemoglobin content in the pooled Matrigel plugs determine by colorimetric assay; means ± SEM, *n* = 3 (right). (**B**) Immunofluorescent staining of sections from Matrigel plug harvested from *Rag1^−/−^* mice after 6 days. B16 tumor cells and *Stat3^+/+^* or *Stat3^−/−^* B cells were mixed with Matrigel then implanted into mice; anti-CD31 (green) and nuclear staining (Hoechst, blue).
(TIF)

Figure S3 ***Stat3*** **in B cells upregulates endothelial cell migration as well as B cell migration.** (**A**) Transwell migration assay to determine the number of endothelial cells (ECs) migrating toward factors released by *Stat3^+/+^* or *Stat3^−/−^* B cells; means ± SEM, *n* = 3. (**B**) B cell intrinsic Stat3 is crucial for B cell migration to the tumor milieu. Transwell migration assay showing the number of tumor-primed *Stat3^+/+^* or *Stat3^−/−^* splenic B cells migrating toward tumor cell-derived soluble factors; means ± SEM, *n* = 4 (left). Representative histograms of CFDA-SE labeling to show proliferation of tumor-primed *Stat3^+/+^* or *Stat3* ^−/−^ splenic B cells upon TCM stimulation. Percentage of proliferative cells were shown as red. Results are representative of 4 independent experiments (right).
(TIF)

Figure S4 **B cells with activated STAT3 accumulate in human tumors and their density in the tumor tissues reflects overall STAT3 activity.** (A) Immunofluorescent staining of many other types of human cancers; anti-CD19 (red; B cell marker) and anti-p-STAT3 (green). Scale bars, 50 µm in the original and 10 µm in the enlarged. (B) Immunofluorescent staining showing CD19- and p-STAT3 (green) and nuclei (DAPI, blue). Scale bars, 10 µm.
(TIF)

Figure S5 **B cells accumulate around microvessels in human prostate tumors.** IHC images showing the accumulation of CD19-positive B cells in human prostate tumor tissues; H&E staining of the consecutive tissue sections.
(TIF)
