# Supplementary figures and images for: B Cells Promote Tumor Progression via STAT3 Regulated-Angiogenesis
Source: PLoS One. 2013 May 29;8(5):e64159. doi: 10.1371/journal.pone.0064159 (PMC3667024; doi:10.1371/journal.pone.0064159)

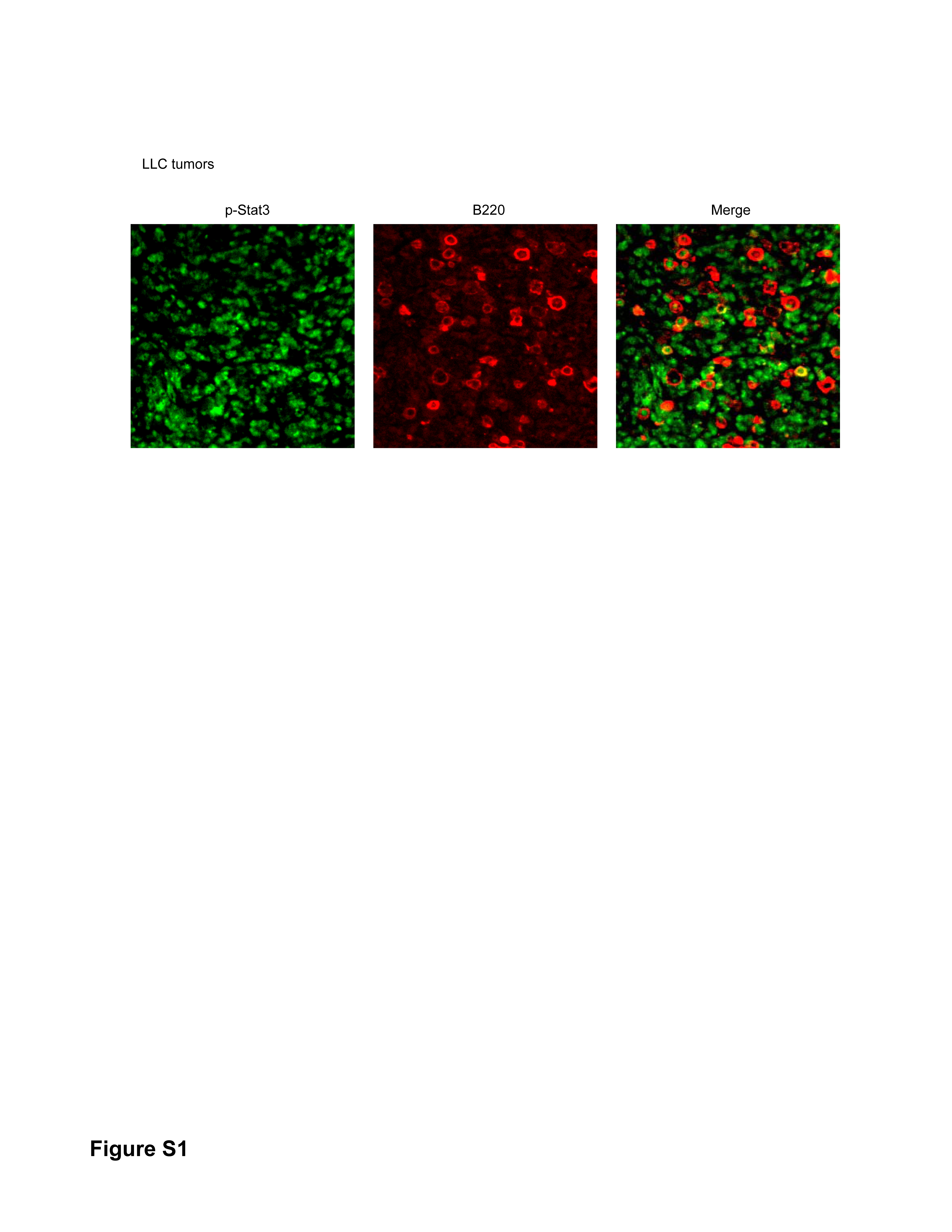

Supplement: Figure S1 — Stat3 is persistently activated in tumor-infiltrating B cells. Immunofluorescent staining of LLC tumors showing p-Stat3-positive cells (green) and B cells (red). LLC tumors were grown in C57BL/6 mice. Tumor-infiltrating B cells were detected with anti-B220 antibodies. (TIF) [file pone.0064159.s001.tif]

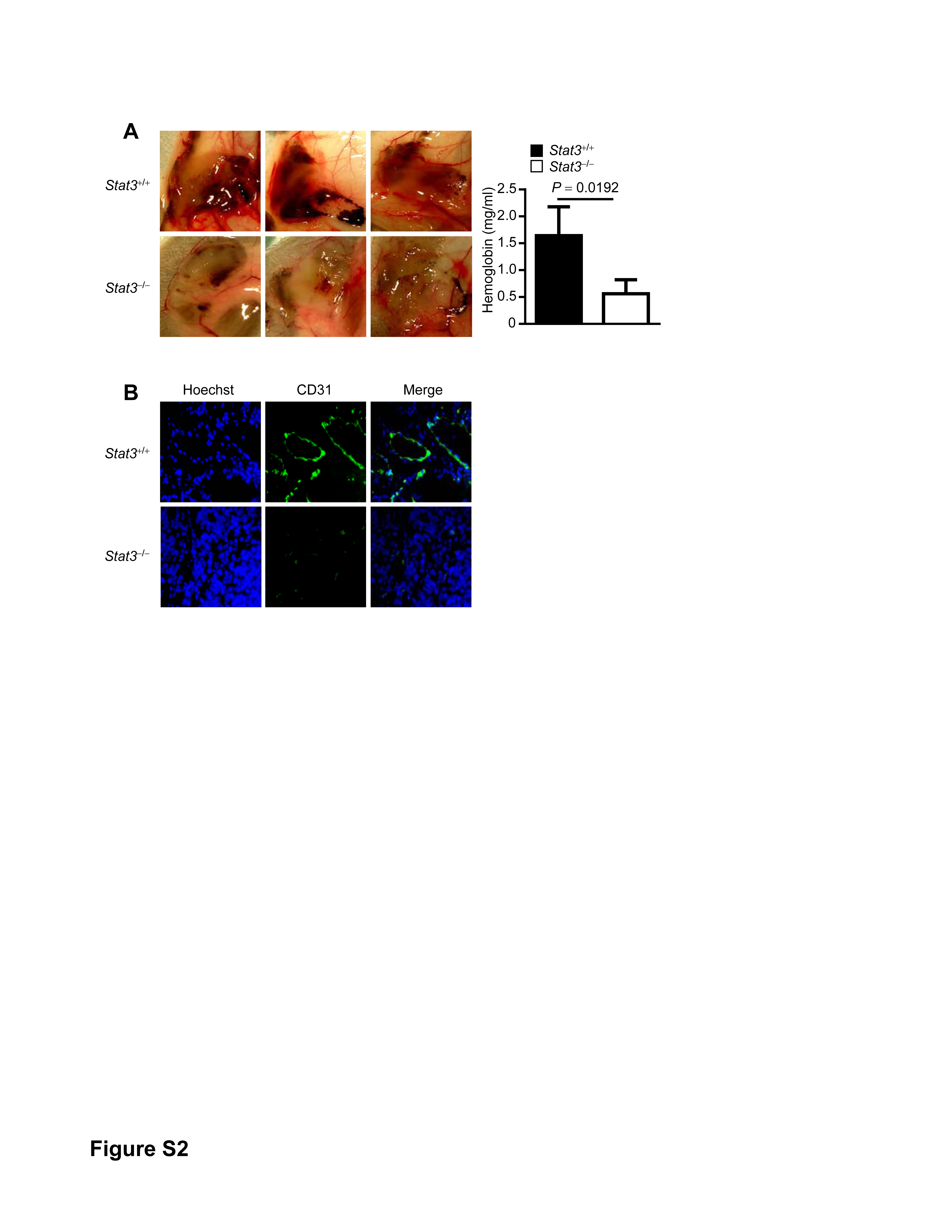

Supplement: Figure S2 — B cells promote tumor angiogeneis in a Stat3-dependent manner. (A) Images of vessel formation in Matrigel pugs containing B16 tumor cells and Stat3+/+ or Stat3−/− B cells; n = 7 (left). Hemoglobin content in the pooled Matrigel plugs determine by colorimetric assay; means ± SEM, n = 3 (right). (B) Immunofluorescent staining of sections from Matrigel plug harvested from Rag1−/− mice after 6 days. B16 tumor cells and Stat3+/+ or Stat3−/− B cells were mixed with Matrigel then implanted into mice; anti-CD31 (green) and nuclear staining (Hoechst, blue). (TIF) [file pone.0064159.s002.tif]

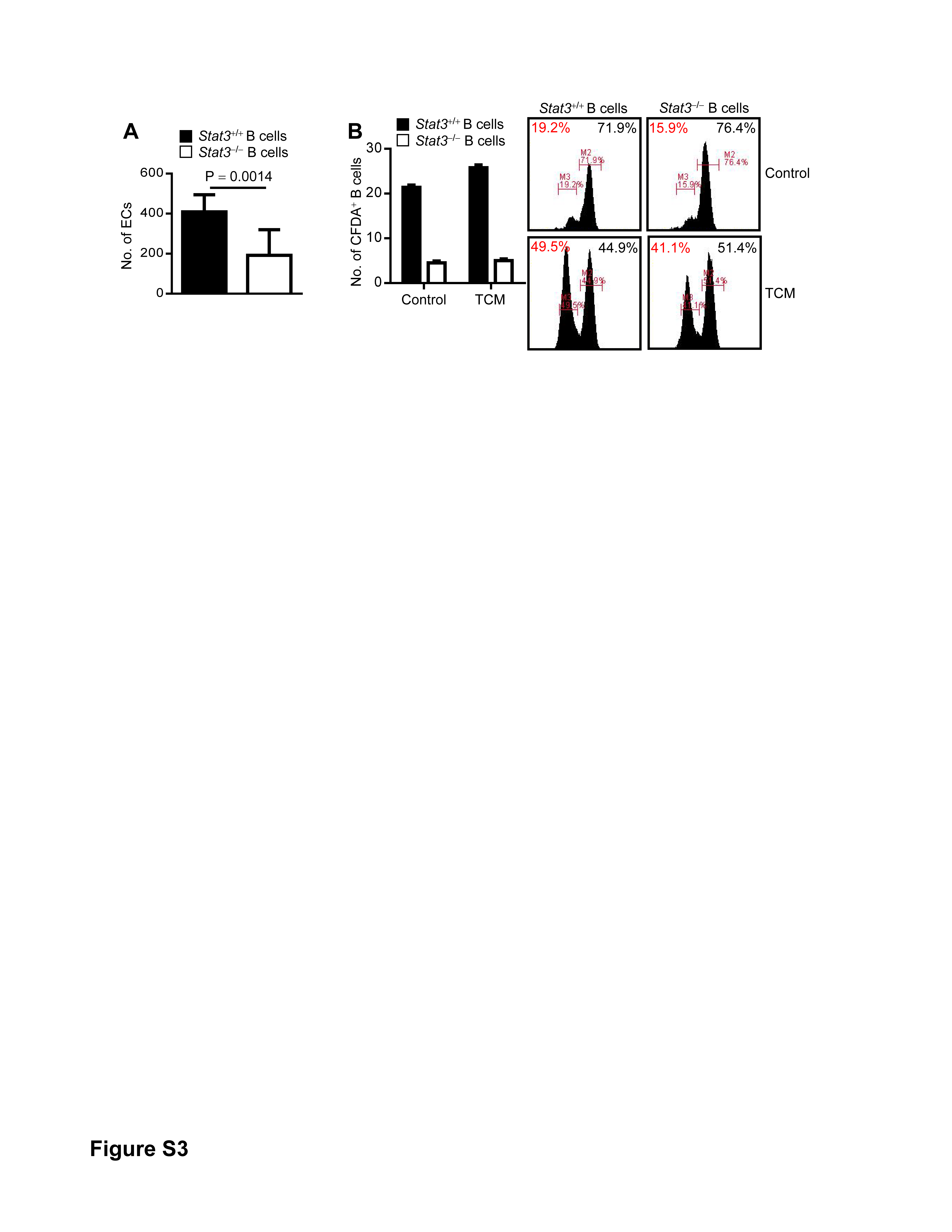

Supplement: Figure S3 — Stat3 in B cells upregulates endothelial cell migration as well as B cell migration. (A) Transwell migration assay to determine the number of endothelial cells (ECs) migrating toward factors released by Stat3+/+ or Stat3−/− B cells; means ± SEM, n = 3. (B) B cell intrinsic Stat3 is crucial for B cell migration to the tumor milieu. Transwell migration assay showing the number of tumor-primed Stat3+/+ or Stat3−/− splenic B cells migrating toward tumor cell-derived soluble factors; means ± SEM, n = 4 (left). Representative histograms of CFDA-SE labeling to show proliferation of tumor-primed Stat3+/+ or Stat3 −/− splenic B cells upon TCM stimulation. Percentage of proliferative cells were shown as red. Results are representative of 4 independent experiments (right). (TIF) [file pone.0064159.s003.tif]

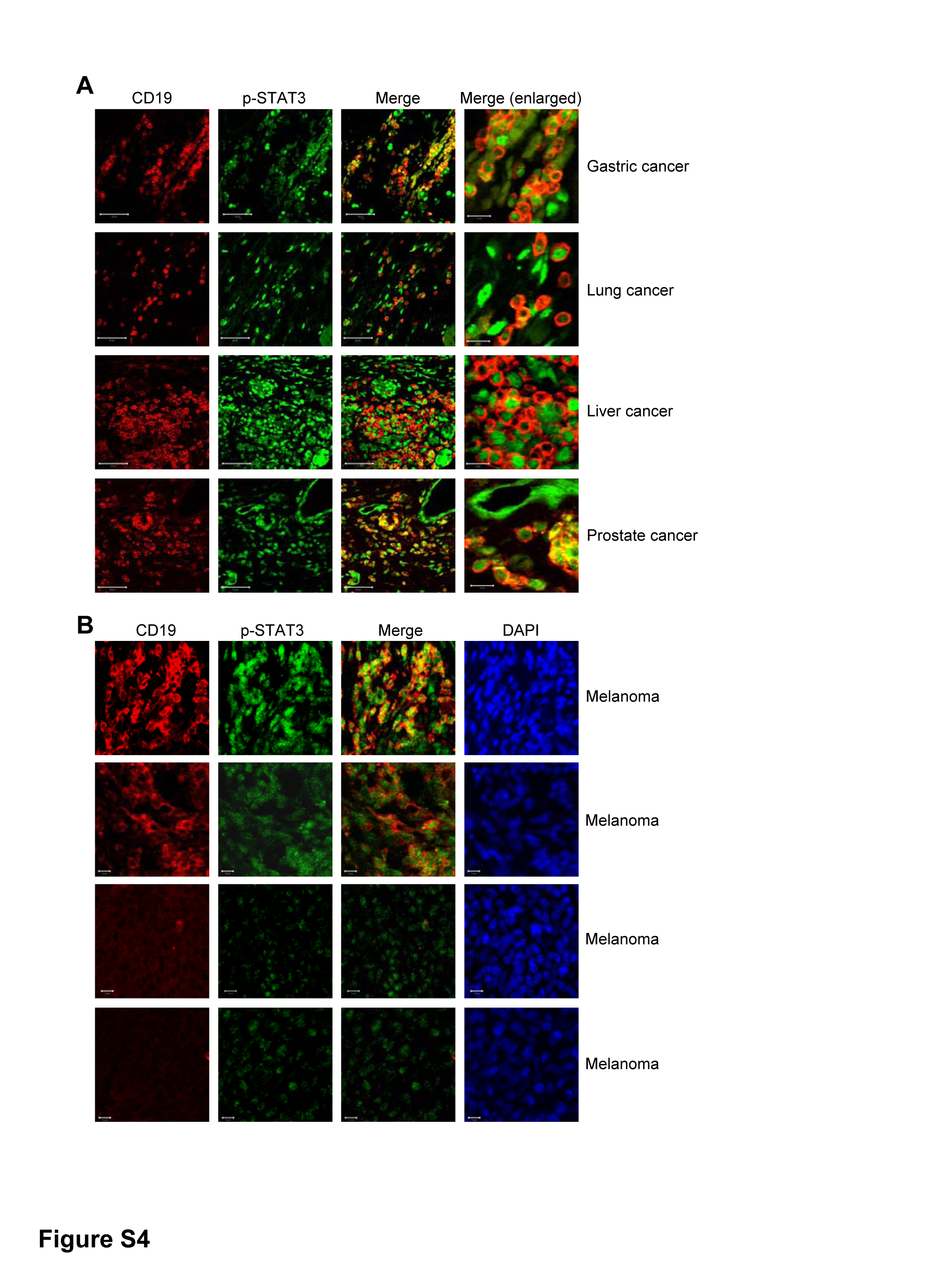

Supplement: Figure S4 — B cells with activated STAT3 accumulate in human tumors and their density in the tumor tissues reflects overall STAT3 activity. (A) Immunofluorescent staining of many other types of human cancers; anti-CD19 (red; B cell marker) and anti-p-STAT3 (green). Scale bars, 50 µm in the original and 10 µm in the enlarged. (B) Immunofluorescent staining showing CD19- and p-STAT3 (green) and nuclei (DAPI, blue). Scale bars, 10 µm. (TIF) [file pone.0064159.s004.tif]

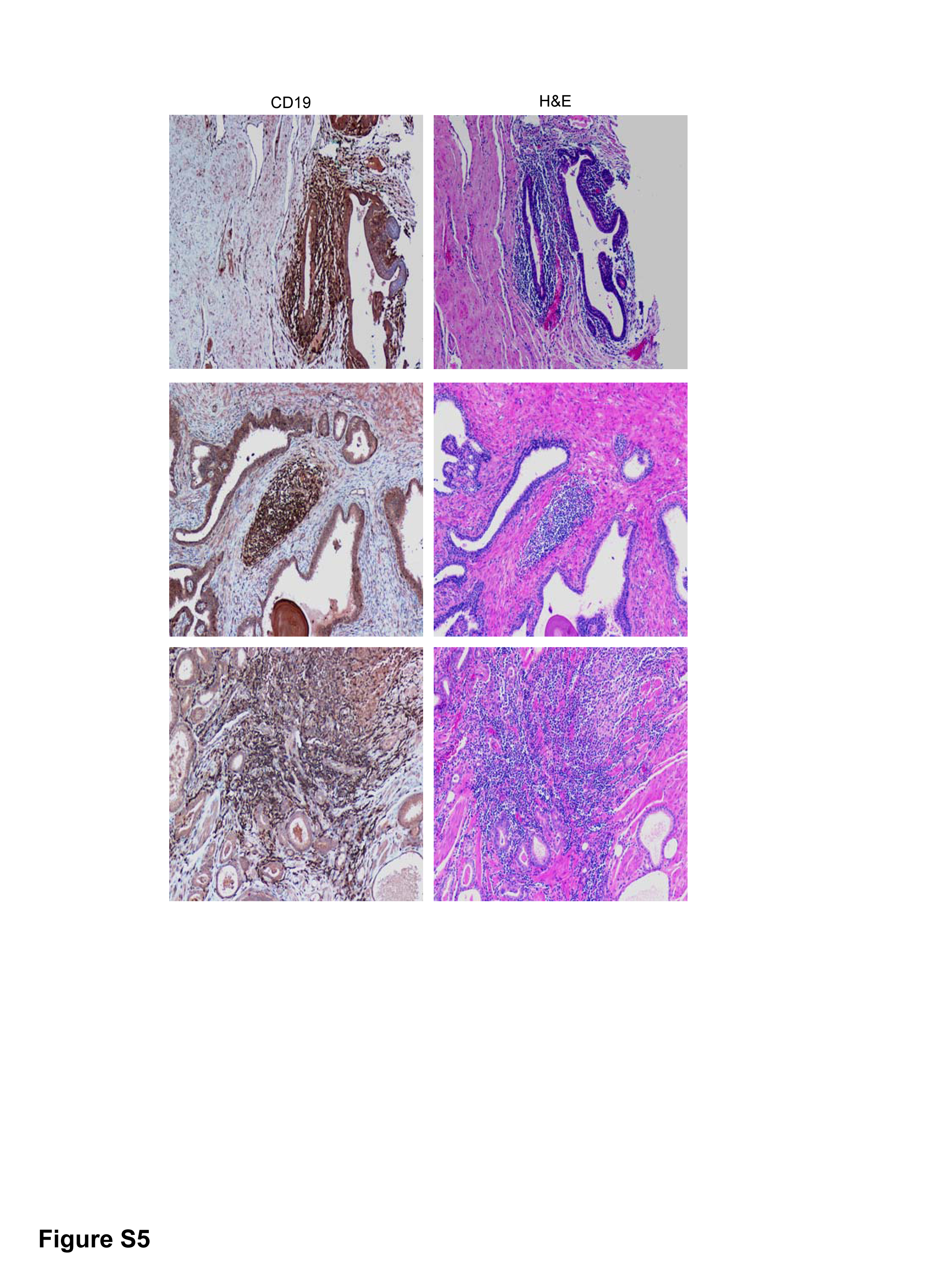

Supplement: Figure S5 — B cells accumulate around microvessels in human prostate tumors. IHC images showing the accumulation of CD19-positive B cells in human prostate tumor tissues; H&E staining of the consecutive tissue sections. (TIF) [file pone.0064159.s005.tif]
